# Isl Identifies the Extraembryonic Mesodermal/Allantois Progenitors and is Required for Placenta Morphogenesis and Vasculature Formation

**DOI:** 10.1002/advs.202400238

**Published:** 2024-06-25

**Authors:** Zeyue Zhu, Qicheng Zou, Chunxiao Wang, Dixi Li, Yan Yang, Ying Xiao, Yao Jin, Jie Yan, Lina Luo, Yunfu Sun, Xingqun Liang

**Affiliations:** ^1^ Key Laboratory of Arrhythmia of the Ministry of Education of China East Hospital Tongji University School of Medicine Shanghai 200120 China; ^2^ Department of Hematology, Tongji Hospital Tongji University School of Medicine Shanghai 200120 China; ^3^ Shanghai East Hospital Tongji University School of Medicine 150 Jimo Road Shanghai 200120 China

**Keywords:** allantois, extraembryonic mesoderm, lineage, Isl1, pregnant maintenance vasculature

## Abstract

The placenta links feto‐maternal circulation for exchanges of nutrients, gases, and metabolic wastes between the fetus and mother, being essential for pregnancy process and maintenance. The allantois and mesodermal components of amnion, chorion, and yolk sac are derived from extraembryonic mesoderm (Ex‐Mes), however, the mechanisms contributing to distinct components of the placenta and regulation the interactions between allantois and epithelium during chorioallantoic fusion and labyrinth formation remains unclear. Isl1 is expressed in progenitors of the Ex‐Mes and allantois the Isl1 mut mouse line is analyzed to investigate contribution of Isl1^+^ Ex‐Mes / allantoic progenitors to cells of the allantois and placenta. This study shows that Isl1 identifies the Ex‐Mes progenitors for endothelial and vascular smooth muscle cells, and most of the mesenchymal cells of the placenta and umbilical cord. Deletion of Isl1 causes defects in allantois growth, chorioallantoic fusion, and placenta vessel morphogenesis. RNA‐seq and CUT&Tag analyses revealed that Isl1 promotes allantoic endothelial, inhibits mesenchymal cell differentiation, and allantoic signals regulated by Isl1 mediating the inductive interactions between the allantois and chorion critical for chorionic epithelium differentiation, villous formation, and labyrinth angiogenesis. This study above reveals that Isl1 plays roles in regulating multiple genetic and epigenetic pathways of vascular morphogenesis, provides the insight into the mechanisms for placental formation, highlighting the necessity of Isl1 for placenta formation/pregnant maintenance.

## Graphical abstract

The diagram showing phenotypes observe in Isl1 mutant allantois and potential underlying mechanisms. Mmp9: Matrix metalloproteinase 9; Mesp1: Cardiovascular development mesodermal‐associated protein 1; Otx2: Orthodenticle homeobox 2; Col3a1: Collagen 3 alpha‐1; Postn: Postsynaptic protein; Lgals3: Advanced glycation end‐product receptor 3; Sox2: High mobility group box protein 2; Zic2: Zic family member 2; Thy1: Thy‐1 cell surface antigen; Pdpn: Podoplanin; Lamc2: Laminin subunit gamma‐2; Eras: ES cell‐expressed Ras; Hesx1: Hesx homeobox 1.

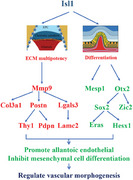

## 1 Introduction

The placenta is the first organ formed during pregnancy and is responsible for the exchange of nutrients, hormones, gases, and waste between the fetus and mother crucial for the success of term pregnancy. Placental malformation and insufficiency lead to pregnancy‐related complications, such as abortion.^[^ ^1^, ^2^, ^3^, ^4^, ^5^ ^]^ Genetic screening analyses of the mouse mutant lines have demonstrated that far more genes are required for normal placentation than previously appreciated, and estimated that two‐thirds of embryonic lethality may be due to placental abnormalities.^[^ ^6^, ^7^ ^]^ However, the underlying molecular mechanisms of the placenta morphogenesis is still unclear.

The placenta is composed of heterogeneous cell types of both maternal and fetal origins. The extraembryonic ectoderm forms the chorionic trophoblast epithelium, while the extraembryonic mesoderm (Ex‐Mes) contributes to the allantois and the mesodermal components of the amnion, yolk sac and chorion. Ex‐Mes cells differentiate into epithelial‐like allantoic progenitors located to the allantois core domain (ACD) and at the boundary between posterior primitive streak and allantois.^[^ ^8^, ^9^ ^]^ ACD progenitors are multipotent and contribute to the vascular and mesenchymal lineages of the allantois.^[^ ^9^, ^10^, ^11^ ^]^ Consistent with their presumptive cellular identity and differentiation potentials, Ex‐Mes cells preferentially express higher level of genes enriched in angiogenesis, BMP and VEGF signaling pathways and distinct sets of genes involved in cell adhesion, migration, and extracellular matrix organization.^[^ ^12^ ^]^

Within the allantois mesenchymal core, allantois vasculogenesis starts from the distal allantois and proceeds proximally to the allantois base where the allantoic vessels join the fetal circulation.^[^ ^13^, ^14^ ^]^ The endothelial cells coalesce to form endothelial tubes that further undergo lumen formation and remodel to form the mature umbilical vessels.^[^ ^14^, ^15^ ^]^ At the distal end, the allantois attaches and fuses with the chorion, a key step in establishing the labyrinth of the placenta.^[^ ^3^, ^16^ ^]^ Upon chorioallantoic fusion, chorion trophoblast epithelium is promoted to differentiate and undergo epithelial‐mesenchymal transition (EMT), invaginate and form chorionic villi, which is coupled with the penetrance of the allantoic vessels into the villi, and subsequently, the villous trophoblasts and allantois‐derived endothelial cells undergo extensive branching and expansion to form the vascular labyrinth that allows efficient fetal–maternal exchange.^[^ ^3^, ^17^ ^]^ Anterior‐posterior (A‐P) polarity established along the allantois axis is essential for pattern of the allantoic vasculature and central insertion of the umbilical cord onto the placenta.^[^ ^10^, ^18^, ^19^ ^]^ Acquisition of polarity is in coincidence with the emergence of the Ex‐Mes and ACD, and is defined by their distinct Hox gene expression. Recent scRNA‐seq studies have revealed that Ex‐Mes and ACD cells from allantoic base express many Hox genes.^[^ ^12^, ^20^ ^]^ Studies have shown that three 5′*Hoxa* genes (*Hoxa10, 11, 13*) are expressed in subsets of Ex‐Mes and allantois vascular progenitor cells and required for placenta vascular patterning and labyrinth vascular formation.^[^ ^21^, ^22^ ^]^

Chorioallantoic fusion is a complex process, requiring proper development of the allantois and chorion, and complex signaling and cellular interactions between different tissues and cell types. A number of transcription factors and signals have been shown to be essential for control of allantois and placental development, and alterations of these genes and signaling pathways cause defects in allantois growth, chorioallantoic fusion, and labyrinth angiogenesis.^[^ ^15^ ^]^ Binding of Vcam1 with its receptor α4‐integrin, which are expressed in the distal allantois and chorion respectively, mediates the chorioallantoic attachment.^[^ ^23^, ^24^ ^]^ Formation of the allantois and fetoplacental vasculature is regulated by many of the same signaling pathways that control embryonic vascular development, such as BMP, FGF, TGFβ, Notch, and PDGF VEGF and their receptors/coreceptors. Several transcription factors, metabolic and epigenetic factors in different cells of the placenta are required for placental development, such as T, Tbx4, Foxf1, Tead4, Cdx2, Gcm1, Esrrb, and Tfap2.^[^ ^1^, ^15^, ^25^ ^]^

The LIM‐Homeodomain transcription factor Isl1 is expressed in diverse tissues, which is mostly derived from the mesoderm during embryonic development and plays essential roles in cell proliferation, differentiation, and survival.^[^ ^26^, ^27^, ^28^, ^29^ ^]^ Isl1 is expressed in the progenitors of pharyngeal cardiac mesoderm and is required for their contribution to the heart.^[^ ^27^ ^]^ Isl1 plays a critical role in sympathetic neuronal proliferation, differentiation, and diversification.^[^ ^30^ ^]^ A recent study in primates has found that Isl1 is expressed in the amnion, where it regulates the amnion signals (BMP4) essential for mesoderm formation, and mutation of Isl1 causes a failure in mesoderm formation.^[^ ^31^ ^]^ In the previous study, we found that Isl1 is expressed in the allantois and surrounding mesenchyme, but the role of Isl1 in allantois and placenta development is unknown.^[^ ^32^, ^33^ ^]^ Here, we have found by immunostaining and lineage tracing that Isl1 identifies a population of extraembryonic mesodermal and allantoic progenitors that contribute to a majority of the vascular and mesenchymal cells of the fetoplacental, and Isl1 is required for these progenitors to contribute to placental development. Isl1 mediates the inductive interactions between the allantois and chorion critical for chorionic epithelium differentiation, villous formation, and labyrinth angiogenesis.

## 2 Results

### 2.1 Isl1 Marks the Ex‐Mes Progenitors that Contribute to the Allantois/Umbilical Cord and Chorion‐Allantoic Plate of the Placenta

**Figure 1:**
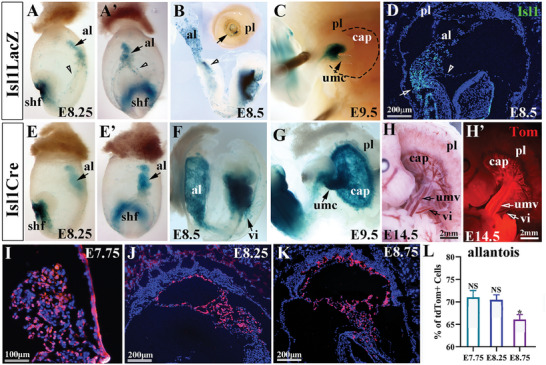
Analysis of Isl1 expression and Isl1 lineages in the allantois and placenta during placenta development. A–C) Wholemount X‐gal staining showing the expression of Isl1‐nLacZ in the allantois (al, arrow)/umbilicus (umc) and surrounding tissues, and the second heart field (shf) from E8.25 to E9.5. a few sporadical X‐gal^+^ cells were found in the amnion and yolk sac (A, A’ arrowhead) D) immunostaining at E8.5 showing a similar pattern of Isl1 expression in the allantois cord domain (ACD) (al, arrow) and proximal allantois. A few sporadical Isl1^+^ cells are also observed in the amnion (arrowhead) E–G) *Isl1* lineage tracing at E8.5‐E9.5 showing Isl1 lineage labeled cells (X‐gal^+^) in the entire allantois (al, arrow)/umbilicus and chorioallantoic plate (cap). (H and H’) The vessels of the umbilical cord (umv) and Chorion plate (cap) are strongly labeled by *Isl1* lineages at E14.5. The proximal vitelline vessels (vi) are also weakly labeled by Is11 lineage but the placenta (pl) is not labeled by Isl1 lineage. I–L) The sections of the allantois of *Isl1*‐Cre; tdTomato samples from E7.75‐E8.75 showing the distribution of Isl1 lineage labeled cells (Tom). (G) Detailed statistical analysis showed that the percentage of Isl1 lineage labeled allantoic cells at E7.75, E8.25, and E8.75 days was 73.5% ± 3.6, 73.7% ± 2.4, and 70.3% ± 2.5, respectively. (n = 5, Error bars represent mean ± SD, 2‐tailed *t*‐test, NS: no statical difference; *p* < 0.05^*^: Statically difference).

Previous studies had shown that Isl1 was expressed in the Ex‐Mes, umbilical cord, and amnion in mice and primates,^[^ ^20^, ^26^, ^31^ ^]^ however the contribution and role of Isl1 during the extraembryonic development were not fully understood. We analyzed Isl1 expression in the Ex‐Mes and its derivatives, and contribution of Isl1 lineage to different cell types of the placenta during development using Isl1‐nLacZ and ‐Cre mice.^[^ ^26^, ^27^, ^33^ ^]^ At E7.5‐8.5, expression of Isl1‐nlacZ was observed in the allantois and Ex‐Mes at the posterior end of the embryo, in addition to the cardiac mesoderm/second heart field (SHF) (**Figure** **1**A). Sporadic Isl1‐nLacZ expressing cells were also detected in the amnion and yolk sac (Figure 1A, A’, B, arrowhead). At E8.5 when allantois attaches and starts to fuse to the chorion, Isl1‐nLacZ expression was gradually downregulated along the proximal‐distal axis of allantois, and only a few Isl1‐nLacZ staining was detected at the site of allantois‐chorionic attachment (Figure 1B, arrow). At E9.0‐9.5, Isl1‐nLazZ expression was confined to the base of the umbilicus (allantois) and surrounding mesenchyme, and no LacZ‐positive cells were detected in the umbilical trunk (Figure 1C, arrow) and the chorioallantoic plate (CAP) of the placenta (Figure 1C, dashed line). Immunostaining at E8.5 with the antibody to Isl1 (Figure 1D) confirmed the expression of endogenous Isl1 in the region of the Ex‐Mes (arrow) and proximal allantois. Sporadic Isl1^+^ cells were also detected in the amnion (Figure 1D, arrowhead), consistent with expression of the Isl1‐nLacZ.

To examine the contribution of Isl1 to the placenta, we performed lineage tracing analysis using Isl1‐Cre and Rosa‐LacZ or ‐tdTomato (Tom) indicator mice. At early stages (E7.5‐E8.5), Isl1 was strongly expressed in the Ex‐Mes and its derived allantois, including the distal part of the allantois that had contacted and partially fused with the chorion (Figure 1E, E’, F). At stages of E7.5‐E9.5, LacZ^+^ positive cells appeared to be in the proximal segments of vitelline vessels (Figure 1F, arrow). At E9.5, in contrast to that of *Isl1*‐nLacZ, *Isl1*‐Cre labeled the whole umbilical cord and chorioallantoic plate, but not the ectodermal‐derived trophoblasts of the placenta (Figure 1G). At later stages of development (E14.5), Isl1 labeled cells (Tom^+^) were found in the whole umbilical cord and chorioallantoic plate vessels, and a relatively weak fluorescent signal was also detected in proximal vitelline vessels of the yolk sac (Figure 1H, H’). Quantification of Isl1 labeled cells (Tom^+^) in the allantoises at distinct developmental stages (E7.5‐E8.75) revealed that the majority of (75.39 ± 3.6% to 65.3 ± 2.47%) allantoic cells were derived from Isl1, statical analysis showed that comparing with Isl1 labeled cells (Tom^+^) in the allantoises at E7.75 and E8.25, that at E8.87 was dramatically decreased (Figure 1I–L, ^*^ marked). Together, our studies suggested that Isl1 labeled a major subset of the progenitors of the Ex‐Mes that contributed to the allantois/umbilical cord and the mesodermal components of the placenta. Similar to its expression pattern in the cardiac mesoderm,^[^ ^33^ ^]^ when Isl1^+^ Ex‐Mes precursors migrated into allantois and differentiate, and Isl1 expression was downregulated later, suggesting Isl1 was a marker for the Ex‐Mes progenitors.

### 2.2 Isl1^+^ Ex‐Mes Progenitors are Multipotent, Can Differentiate into Multiple Cell Types of the Placenta

**Figure 2:**
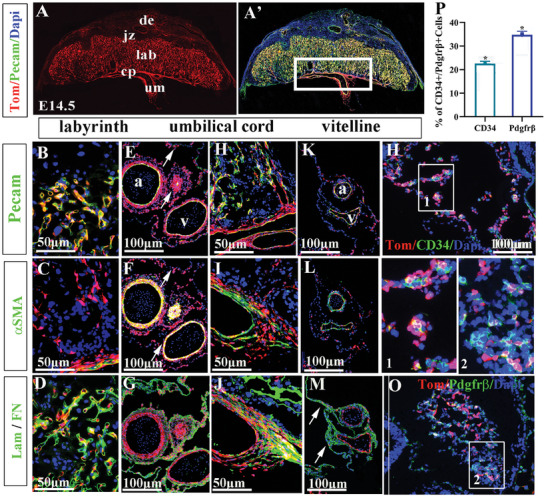
Contributions of Isl1 lineage to the allantois/umbilicus and placenta analyzed by *Isl1* lineage tracing and coimmuostaining. A, A’) A whole mount view of a *Isl1*‐Cre; Rosa‐tdTomato placentas (pl) at E14.5 coimmunostained with Pecam (green). Coimmunostaining was performed with antibodies to Pecam (B, E, H, K), αSMA (C, F, I, L), Laminin (Lam, D) and Fibronectin (G, J, M) to label endothelial cells, smooth muscle, fibroblasts and extracellular matrix, respectively. B–M) showing the contributions of Isl1 lineage (Tom+) to distinct cell types in labyrinth (B‐D), umbilicus (E‐G), chorion plate (H‐J) and vitelline vessels and yolk sac (K‐M). N–P) Coimmunostaining at E8.5 with antibodies to CD34 (N) and Pdgfrβ (O) to mark endothelial and mesenchymal progenitors, respectively. a: artery; cap: chorion plate; de: decidua; jz: junctional zone; lab: labyrinth; v: vein; umc: umbilical cord; vi: vitelline vessel. (n = 5, Error bars represent mean ± SD, 2‐tailed *t*‐test, *p* < 0.05^*^, Statically difference).

To assess the differentiation potential and contribution of Isl1^+^ Ex‐Mes progenitors to cell types of the placenta (including the umbilicus) during developmental stages, we performed Isl1 coimmunostaining with antibodies and lineage tracing that mark different cell types of the vasculature and mesenchyme. Analyses were performed on the placenta at E14.5 when the placenta is fully matured morphologically and functionally. A representative cross‐section of the placenta showed *Isl1*‐Cre labeled vascular endothelial cells (Pecam^+^/Tom^+^) in the labyrinth and umbilical cord (**Figure** **2**A, A’). Detailed analyses revealed that all the endothelial cells (Pecam^+^) of the labyrinth and umbilical/chorioallantoic plate, and the proximal trunk of the vitelline vessels were labeled by *Isl1*‐Cre (Tom^+^) (Figure 2B,E,H,K). Labyrinth vessels were outlined by the expression of base membrane marker laminin (Lam) and did not express smooth muscle marker αSMA (Figure 2C,D). *Isl1*‐Cre labeled cells contributed to nearly all the vascular smooth muscle cells (VSMCs) (αSMA^+^, 97.94 ± 1.9%, n = 5) and mural/perivascular mesenchymal cells or fibroblasts (Fibronectin, Fn^+^, 97.39 ± 2.46%, n = 5) of the umbilical cord (Figure 2F,G). Substantial subsets of VSMCs and mural/perivascular mesenchymal cells in the chorioallantoic plate were also labeled by Isl1 (Figure 2H–J). Consistent with previous studies, we observed a substantial subset of cells in the amnion membrane (Fn^+^) associated with the umbilicus are labeled by *Isl1*‐Cre (Tom^+^) (Figure 2E–G, arrow) (58.47% ± 4.33, n = 5). In contrast, only a small subset of vitelline VSMCs (13.45 ± 3.02%, n = 5) and yolk sac membrane cells (Fn^+^) (16.27% ± 1.51, n = 5) were labeled by Isl1 (Figure 2L,M). Immunostaining of the allantois at E8.25 with antibodies to the progenitors of endothelial (CD34) and mesenchymal cells (Pdgfrβ) revealed clusters of Isl1 labeled allantoic cells (Tom^+^) that co‐expressed CD34 (Figure 2N,P, boxed inlet 1) or Pdgfrβ, statical analysis showed that it was significant (Figure 2O,P, ^*^ *p* < 0.05, boxed inlet 2). Results demonstrated that Isl1^+^ Ex‐Mes progenitors were multipotent and differentiated into placental (umbilical) endothelial cells, VSMCs, and mesenchymal cells.

### 2.3 Isl1 Deficiency Caused Defects in Allantois Growth, Chroioallantoic Fusion, and Placenta Vasculogenesis

**Figure 3:**
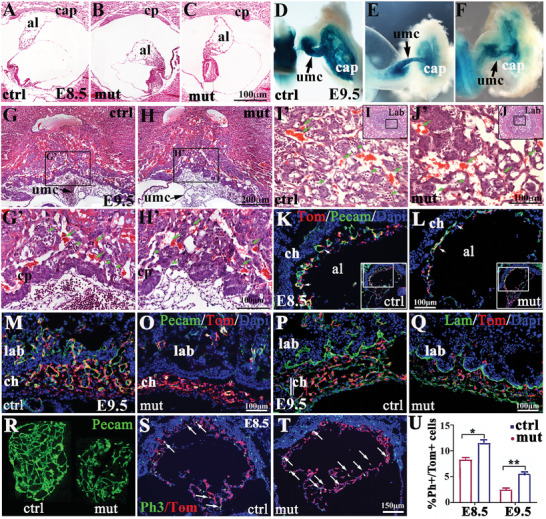
Essential of Isl1 in the allantois growth and vasculogenesis, chorioallantoic fusion and labyrinth formation. A–C) Histological analyses showing defects in allantois growth and a lack of chorioallantoic fusion in *Isl1* mutants compared to control littermates at E8.5. D–F) At E9.5, X‐gal staining showing the swollen/hydropic and slender umbilical cord (umc; arrows) and smaller or irregular shaped fetoplacenta in *Isl1* mutant embryos. G–J) Histolgical analyses showing the labyrinth morphogenesis of *Isl1* mutants and littermate controls at E9.5. The arrow and arrowhead pointing to the maternal and fetal red blood cells, respectively). K,L) Pecam immunostaining at day E8.5 showing impaired chorioallantoic fusion and vasculogenesis in the allantois of *Isl1* mutants. M–Q) Pecam and laminin immunostaining showing impaired labyrinth branching morphogenesis of *Isl1* mutants at E9.5 compared to that of the controls. R) Impaired angiogenesis in explant culture of the allantoises isolated from E8.25 *Isl1* mutants and control embryos, samples stained with Pecam antibody. S–U) PH3 immunostaining and quantification showing significantly increased proliferation of allantois's cells in *Isl1* mutants at E8.5 and E9.5 compared to control littermates by detailed statically analysis. (n = 5; Error bars represent mean ± SD, 2‐tailed *t*‐test, *p* < 0.05^*^ and *p* < 0.01^**^). cp: chorion plate; al: allantois; cap: chorioallantoic plate; lab: labyrinth; ch: chorion; umc: umbilical cord.

Placenta was a vascular organ and placental circulation was essential for fetal growth and survival. Previous studies had shown that *Isl1* knockout mice die at round E9.5‐10.5 due to cardiac defects.^[^ ^27^, ^34^ ^]^ Given the prominent expression of Isl1 in the progenitors of mesodermal components of the placenta and the major contribution of Isl1 lineages to placenta (umbilical cord) vasculature, we speculated that Isl1 might play a critical role in the establishment of placenta circulation. We crossed *Isl1*‐Cre mice (*Isl1* ^Cre/+^) with heterozygous floxed Isl1 mice (*Isl1* ^f/+^) and examined the growth and survival of *Isl1* mutant embryos (*Isl1* ^Cre/f^) at early developmental stages. Genotyping revealed that the number of *Isl1* mutants (*Isl1* ^Cre/+^) from E8.5 to E11.5 was gradually decreased (**Table** **1**), consistent with previous reports on *Isl1* global knockout mice.^[^ ^35^, ^36^ ^]^ Interestingly, we observed a nearly 32–35% reduction in the expected number of *Isl1* mutant embryos at E8.5, the morphology of which appeared normal before the heart was formed and functional. Wholemount examination revealed that a number of embryos at E8.5 and E9.5 exhibited defective allantois growth and a failure to undergo chorioallantoic fusion, which may account for early embryonic lethality in *Isl1* mutants. We further examined the allantois and placenta development of *Isl1* mutants (*Isl1* ^Cre/f^) and littermate controls at E8.5 and E9.5 by wholemount X‐gal staining and histological analysis. At E8.5, the allantois of littermate controls had attached and fused with the chorion, whereas *Isl1* mutants exhibited variable degree of defects in allantois growth, attachment, and fusion (**Figure** **3**A–C). Wholemount X‐gal staining showed that at E9.5 the allantois of control embryos had grown and remodeled into the umbilical cord and the chorioallantoic plate had been well established. Both the umbilical cord and chorioallantoic plate are strongly labeled by Isl1. In about 27% of *Isl1* mutant embryos, the allantois and chorion failed to undergo chorioallantoic attachment and fusion. However, the remaining *Isl1* mutant embryos, in which fusion between the allantois and the chorion occurred, displayed variable allantoic defects, including swollen/hydropic or slender umbilical cord, and smaller or irregular shaped chorioallantoic plate. Intensity of X‐gal staining of *Isl1* mutant umbilical cord and the chorioallantoic plate was markedly reduced (Figure 3D–F). We further analyzed the chorioallantoic and labyrinth morphogenesis and placenta vasculogenesis in *Isl1* mutants. Histological analyses of cross‐section of placentas at E9.5 (Figure 3G, H‐, I, J‐coronal section) revealed that the control allantois had remodeled into a central umbilical vessel and chorioallantoic vascular plexus that were filled with fetal nucleated red blood cells (fnRBCs). The allantoic vessels had penetrated the chorion plate, and extensive labyrinth vessels were observed that are filled with fnRBCs (arrowhead) and separated with maternal circulation (arrow) by a thin layer of placenta barrier, suggesting that the placenta circulation was functional (Figure 3G, G’, I, I’). In contrast, in *Isl1* mutants, remodeling of allantois into the central umbilical blood vessel was impaired, and allantoic vessels failed to penetrate the chorion plate, branch, and expand, suggesting impaired vasculogenesis and angiogenesis, few if any fnRBCs were seen in fetal vessels (arrowhead). The chorion plate of *Isl1* mutants appeared to be thicker and clumping, suggesting impaired trophoblast differentiation and villous formation, which may lead in part to impaired blood vessel penetration, and labyrinth formation observed in *Isl1* mutants (Figure 3H, H’, J, J’). We further examined placental vasculogenesis in *Isl1* mutants at early developmental stages by immunostaining and lineage tracing. At E8.5, the control allantoic vessels (Pecam^+^) migrated toward and started to invade the chorion plate (Figure 3K), and at E9.5 labyrinth vessels branched and expanded extensively (Figure 3M). However, in *Isl1* mutants, the allantoic vessels appeared like primitive endothelial tubes with impaired lumen formation (Figure 3L,O,Q). The mutant vessels were orientated parallel to and did not invade the chorionic epithelium (Figure 3L). The percentage of allantoic endothelial cells (Pecam^+^) relative to Isl1‐derived cells (Tom^+^) was significantly reduced in *Isl1* mutants compared to control littermates (64.25% ± 5.32 versus 38.5% ± 5.45, ^*^, *p* < 0.05, *n* = 5). Fewer mutant vessels at E9.5 were found in the labyrinth that did not appear to branch and expand properly (Figure 3O). Similarly, Laminin immunostaining revealed superficial penetration of *Isl1* mutant allantoic vessels into the chorionic epithelium compared to that of the controls (Figure 3P,Q). In addition, explant cultures of E8.25 allantoises revealed reduced angiogenesis in *Isl1* mutants (Figure 3R). Impaired allantoic vasculogenesis and angoigenesis in *Isl1* mutants could be caused by abnormal allantoic cell proliferation. Phospho‐histone 3 (Ph3) immunostaining showed that the proliferation of Isl1 lineage allantoic cells (Tom^+^) in *Isl1* mutants at E8.5 and E9.5 was significantly increased (Figure 3S–U, arrow), which was show by immunostaining by Ki67 (Figure 5E,F) and cleaved Caspase3 immunofluorescence staining showed that no significant change in cell death was detected in *Isl1* mutant allantois compared to the control, suggesting that the defect in placental vessel formation might be due to impaired differentiation and recruitment of the Ex‐Mes and its derived vascular progenitor cells.

**Table 1:** Genotype and phenotype of offspring from intercrosses between *Isl1* ^Cre/+^ and *Isl1* ^flox/+^ mice.

Collection time	Total embryo and decidua	Genotype				Abnormal embryos	
		*Isl1* ^+/+^	*Isl1* ^Cre/+^	*Isl1* ^f/+^	*Isl1* ^Cre+/f^ (mutant)	Absence of chorioallantoic fusion	Resorbed
E8.5	92	20	23	25	13	4	8
E9.5	76	20	22	20	8	2	4
E10.5	55	18	14	16	4	0	3
E11.5	38	14	11	8	1	0	4

^*^Embryos were died, and nearly or completely resorbed and their genotype could not be reliably determined.

### 2.4 Isl1 is Essential for Maintenance and Differentiation of Ex‐Mes Progenitors During Early Allantois Development

**Figure 4:**
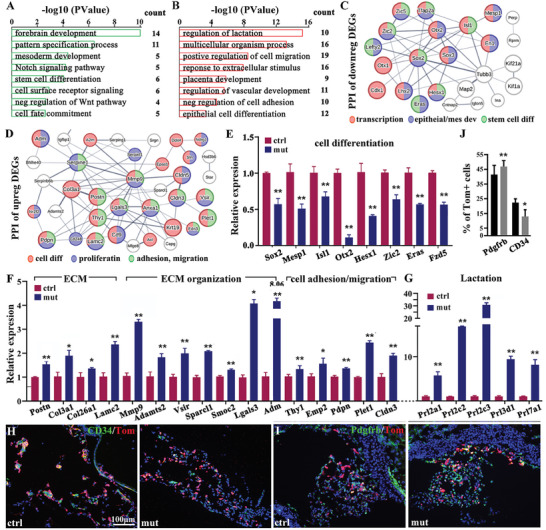
RNA‐seq analyses reveal dysregulation signaling pathways and altered potency and differentiation of Ex‐Mes and allantoic progenitors.  RNA‐seq analyses verified that the top enriched GO terms for downregulated genes were required for the forebrain formation, pattern specification, mesoderm development, stem cell differentiation and cell fate commitment and cell surface, and negative regulation of Wnt signaling pathwayGO analysis. (A, B) comparing with ctrl, downregulated and upregulated in mut allantois. String protein‐protein association analysis at E8.25. (C, D). comparing with ctrl, down‐regulated genes in the mutants (C): Color labels: red‐transcription; blue‐epitheial/mes development; green‐stem cell differentiation, up‐regulated genes in the mutants (D) Color labels: red‐cell differentiation; blue‐proliferation; green‐adhesion, migration. qPCR analysis. (E‐G). comparing with ctrl, downregulated genes associated with stem cell differentiation and mesoderm development in the mutants, upregulated genes involved in ECM, ECM organization, prolactin and cell adhesion and migration in mut. (values are normalized by GAPDH expression level and indicated as mean ± SD. N = 5. ^*^ *p* < 0.05, ^**^ *p* < 0.01). Immunostaining at E8.5. (H‐J). comparing with ctrl, decreased endothelial progenitors (CD34^+^), and increased mesenchymal cells (Pdgfrβ+) in the mut. Statistical analysis. J). At day E8.5, a decrease in the number of CD34+ endothelial progenitors and an increase in Pdgfrβ+ mesenchymal cells was significantly different in the mut (mean ± SD, n = 5, ^*^ representing *p* < 0.05, and bilateral *t*‐test).

To investigate the molecular mechanisms underlying the role of Isl1 in the Ex‐Mes and allantois morphogenesis, we performed RNA‐seq analyses of allantoises of *Isl1* mutants (*Isl1* ^Cre/f^; Rosa‐Tomato) and control littermates (*Isl1* ^Cre/+^;Rosa‐Tomato) at E8.25. Samples enriched in Ex‐Mes and allantoic progenitor cells were dissected under fluorescence microscope from the allantoises, including allantoic core domain (ACD) and adjacent Isl1^+^ extraembryonic tissues. RNA‐seq analysis identified 209 differentially expressed genes (DEGs) in *Isl1* mutants (77 downregulated and 132 upregulated) (|fold change Isl1‐mut versus ctrl | ≥ 1.3, *p* < 0.05) (Table S2, Supporting Information). Gene ontology (GO) analysis revealed that the top enriched GO terms for downregulated genes were required for the forebrain formation, pattern specification, mesoderm development, stem cell differentiation, and cell fate commitment. In addition, Notch and cell surface receptor signaling pathways, negative regulation of Wnt signaling pathway (**Figure** **4**A; Table S2, Supporting Information). For upregulated genes, overrepresented categories included regulation of placenta development, lactation, multicellular organism process, cell migration and adhesion, response to extracellular stimulus, and guideline of vascular development and epithelial cell differentiation (Figure 4B; Table S2, Supporting Information). These data suggested deletion of *Isl1* resulted in dysregulated signaling pathways and altered potency and differentiation of Ex‐Mes and allantoic progenitors. STRING protein‐protein association analysis was performed on down‐ and up‐regulated genes in *Isl1* mutants at E8.25, and GO analysis was simultaneously applied to the network proteins to better understand the protein‐protein interactions (PPI) and the pathways and networks regulated by Isl1. The nodes (genes) were colored by their enriched terms and the size of the selected key nodes was enlarged for better visualization. The thickness of the edge indicates the **confidence** level **of** the **interaction**. Those nodes that do not interact with the main network were filtered out (Figure 4C,D). String analysis of downregulated genes revealed two interconnected clusters. The first cluster contained a number of transcription regulators (red) important for the mesoderm development (blue) (*Sox2, Lhx2, Mesp1, Otx2, Zic2, Lefty2*, and *Cdx1*) and stem cell differentiation (green) (*Sox2, Hesx1, Mesp1, Isl1, Tfap2a, Zic2/5*, and *Lefty2*) (Figure S1, Supporting Information). The second cluster included genes of microtubule structure and organization (*Tubb3, Map2, Kif21a, Kif1a, Ina*). Isl1 interacted with other transcription regulators (*Mesp1, Sox2, Sox3, Otx2, Tfap2a*, and *En1*), and might be required for the maintenance of the network‐associated gene expression, and in the absence of *Isl1*, those expressions were downregulated. In addition, Isl1 interacted directly or indirectly with microtubule genes (*Tubb3* and *Map2*) important for cell differentiation and motility. Selected DEGs downregulated in *Isl1* mutants were verified by qPCR, including those involved in stem cell differentiation and mesoderm development (*Sox2, Mesp1, Isl1, Otx2, Hesx1, Zic2, Eras*, and *Fzd5*) (Figure 4E). For upregulated DEGs, String analysis revealed a main PPI network consisting of several subclusters of proteins involved in ECM organization (e.g., *Col3a1, Col26a1, Lamc2, Postn, Mmp9, Adamts2, Vsir, Smoc2, Sparcl1*, and *Adm*), cell adhesion and migration (e.g., *Thy‐1, Emp2, Pdpn, Cldn3/5, CD9, Lgals3, Krt19, Plet1*, and *Procr*), protease bind and inhibitor activities (e.g., *Serpins, A2m*), regulation of vasculature and angiogenesis (e.g., *Col3a1, Mmp9, Thy1, Lgals3, Pdpn, Cldn5, Serpine1/f1, Nr2f2*, and *Epas1*) and stem cell differentiation (e.g., *Tfap2c, Dnmt3l, Vsir, Edn3*, and *A2m*) (Figure 4D). Prolactin genes are widely expressed at the fetal‐maternal interface, including umbilical mesenchyme and endothelium, important for cell proliferation, migration, and female reproduction.^[^ ^37^, ^38^, ^39^ ^]^ We observed that a subcluster of prolactin family members and related synthesis enzymes were upregulated that may account in part for the increased proliferation observed in *Isl1* mutant allantois (Figure 4K). Increased mRNA expression of selected genes enriched in ECM, ECM organization, and cell adhesion and migration, and prolactin were verified by qPCR (Figure 4F,G). In addition, the mammalian phenotype ontology analysis on the DEGs of *Isl1* mutants at E8.25 identified a number of genes implicated in abnormal extraembryonic tissue morphology, including *Tfap2c, Utf1, Dnmt3l, Adm, Nr2f2, Epas1, F3, Procr, Prl4a1*, and *Prl8a2* (Figure 4K). Marked increase in ECM related genes observed in *Isl1* mutant allantoises suggested the increased number of differentiated mesenchymal cells and ECM producing cells. We performed immunostaining to examine the differentiation of endothelial progenitors (CD34^+^) and mesenchymal cells (Pdgfrβ^+^) at E8.5. We found a significant decrease in the number of CD34^+^ endothelial progenitors but an increase in Pdgfrβ^+^ mesenchymal cells in *Isl1* mutant allantoises (Figure 4H–J). Together, our studies demonstrated that Isl1 plays a critical role in the maintenance of Ex‐Mes progenitor cell potency. Despite that Isl1 expression is diminished as the EX‐Mes progenitor cells differentiate, a transient expression of Isl1 in allantoic progenitors appears to be required for promoting endothelial but repressing mesenchymal cell differentiation.

### 2.5 Isl1 is Essential for Placental Vasculature Morphogenesis and Inductive Interactions Important for Chorion Epithelial Differentiation and Labyrinth Formation

**Figure 5:**
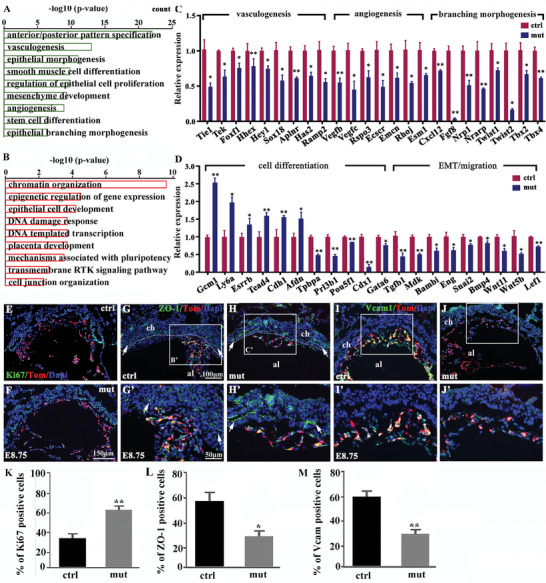
Isl1 is essential for placental vasculature morphogenesis and the interactions important for chorion epithelial differentiation and labyrinth formation. A,B) GO analyses showing the top enriched terms for upregulated and downregulated genes in *Isl1* mutant allantoises and fetoplacentas. C,D) qPCR validation of selected genes involved in vasculogenesis, angiogenesis, morphogenesis, cell differentiation, EMT and cell migration, the interactions between networks of the up and downregulated DEGs by importing DEGs involved in extraembryonic tissue morphogenesis into the String database. Color labels: green‐transcriptional and signaling pathways; red‐epithelial cell development. (n = 5, Error bars represent mean ± SD, ^*^ *p* < 0.05, ^**^ *p* < 0.01, 2‐tailed *t*‐test). At E8.75, Ki67 immunostaining and quantification showing increased proliferation in *Isl1* mutant allantois and chorion. E–J) Ki67 immunostaining showing markedly reduced expression of proliferation in the distal allantois of *Isl1* mutants. ZO‐1 immunostaining showing increased and abnormal ZO‐1 protein expression in the chorionic epithelium. Vcam1 immunostaining showing markedly reduced expression of Vcam1 in the distal allantois of *Isl1* mutants. (K‐M) statical analysis showed that changes of Ki67, ZO‐1 and Vcam were significant (n = 5, Error bars represent mean±SD, ^*^ *p* < 0.05, ^**^ *p* < 0.01, 2‐tailed *t*‐test).

To further understand the role of Isl1 in the inductive interactions between the allantois and chorion epithelium during chorioallantoic fusion and labyrinth formation, we performed RNA‐seq analysis of the feto‐placentas and associated allantoises at E8.75. We identified 471 downregulated and 403 upregulated genes in *Isl1* mutants (|fold change Isl1mut versus ctrl | ≥ 1.5, *p* < 0.05) (Table S3, Supporting Information). GO analysis revealed that significantly enriched GO terms for downregulated genes in *Isl1* mutant placenta included anterior‐posterior (**Figure** **5**A–P) pattern specification, vasculogenesis and angiogenesis, signaling, and transcriptional regulation of epithelial morphogenesis and proliferation, smooth muscle cell differentiation, mesenchyme development, stem cell differentiation and epithelial branching morphogenesis (Figure 5A). A‐P polarity established along the allantois axis is essential for patterning the allantoic vasculature and central insertion of umbilical cord into the placenta.^[^ ^10^, ^19^ ^]^ A recent scRNA‐seq study has revealed that Ex‐Mes cells from allantoic base express many Hox genes essential for A‐P specification and patterning.^[^ ^20^ ^]^ Consistent with this, our String analysis revealed a subcluster of downregulated DEGs containing multiple Hox genes and genes critical for A‐P pattern specification and mesoderm development, including transcriptional factors (*T, Msx1, Foxh1, Foxf1, Cdx1*, and *Tbx6*), signaling pathways (*BMP4, FGF8, Lef1, Dkk1, Sfrp1*) and Notch effectors (*Hes1, Hes7, Hey1*, and *Nrarp*) (Figure S1, Supporting Information, colored red). Many of those genes (e.g., *T, BMP4, Cdx1, Foxf1*, and *Lef1*) are known to be expressed in allantois and are required for allantois growth and chorioallantioic fusion.^[^ ^15^ ^]^ In GO term of blood vessel development (Figure S2, Supporting Information, blue), there were several key genes associated with signaling pathways regulating vascular formation (*VEGF/Nrp1, TGFβ, BMP4, Fgf8, Notch, Wnt, Tie1/Tie2, Eng, Cd34, Aplnr, Esm1, and Ecscr*) and transcription factors (*T, Sox18, Sox15, Sox7, Etv2, Hand2, Tbx2, Tbx4, Gata6, Foxf1, Foxh1*, and *Hhex*). A large number of signaling and transcriptional regulators were redundantly involved in controlling of stem cell differentiation (green), mesenchyme (yellow) and epithelium (pink) development, EMT and branching morphogenesis, including *BMP4, Fgf8, Wnt11, Notch, TGFβ1, Twist, Snai2, Eng, Nrp1, Msx1, Sox11, Gata5* and *Tbx2*. We confirmed by qRT‐PCR the alterations in the expression of selected genes that could contribute to the observed vascular and chorionic phenotypes. Those included genes involved in the vasculogenesis (*Tie1, Tek, Foxf1, Hhex, Hey1, Sox18, Aplnr, Has2*, and *Ramp2*), angiogenesis (*Vegfb, Vegfc, Rspo3, Ecscr, Emcn, Rhoj*, and *Esm1*), branch morphogenesis (*Cxcl12, Fgf8, Nrp1*, and *Nrarp*) and related transcription factors *Tbx2, Tbx4* and its downstream effectors *Twist1, Twist2* (Figure 5C). These results suggested that Isl1 is essential for placental vasculature morphogenesis, and *Isl1* ablation caused impaired chorionic epithelium differentiation, EMT, and labyrinth morphogenesis, consistent with the phenotypes observed in *Isl1* mutants (Figure 3). For upregulated genes in *Isl1* mutants at E8.75, enriched GO terms included chromatin organization, epigenetic regulation of gene expression, epithelial cell development, cell junctional organization, DNA damage response, DNA‐templated transcription, placenta development, mechanisms associated with pluripotency, transmembrane RTK signaling pathways (Figure 5B). Further String analyses on upregulated DEGs in *Isl1* mutants revealed a complex PPI network with a centrally positioned and interconnected cluster comprised of genes enriched in chromatin organization (e.g., *Dnmt1, Dnmt3b, Kat6a/b, Chd2/4/6/8, Setd1b, Setd2, Tet2, Kmt2a, Kdm4a, Kdm5a, Atrx, Arid4a* and *Brca1*) and transcriptional regulation of gene expression (Figure S3, Supporting Information, red and green). Our previous study has revealed a role for Isl1 in chromatin remodeling in cardiac progenitor cells required for cardiac development.^[^ ^34^ ^]^ Together, these studies may suggest the essential role of Isl1 upstream of a complex epigenetic pathway in extraembryonic tissue development. One sub‐network comprised clusters of genes involved in cell‐cell adhesion and epithelial cell development (*Cdh1, Klf5, Afdn, Wt1, IQGAP1, Atrx, Arid4a, Pdpk1, Frs2, Akap9, Fat1*, and *Il6st*) (blue). Cdh1 and Afdn are cell‐cell adhesion molecules essential for epithelial cell development. *Afdn* mutant embryos lacks an allantois.^[^ ^40^ ^]^ Ras GTPase‐activating‐like protein IQGAP1 plays a crucial role in actin cytoskeletal assembly and cell motility. Klf5 is required for suppressing differentiation gene expression in embryonic stem cells and maintained pluripotency.^[^ ^41^ ^]^ Klf5 is preferentially expressed in epithelium of multiple tissues and plays an important role in proliferation and morphogenesis of epithelial cells.^[^ ^42^ ^]^ *Klf5* is also required for the specification and differentiation of extra‐embryonic lineages.^[^ ^43^ ^]^ Both *Atrx* and *Arid4a* are also the key nodes of chromatin organization network and involved in transcriptional regulation and chromatin remodeling. The *Cdh1* subcluster is connected to the core/central epigenetic and transcriptional network via *Cdh1, ATRX*, and *Arid4a*. There was a subcluster of genes (*Esrrb, Gcm1, Tead4*, and *Klf5*) critical for chorionic trophoblast development, which interacted with the *Cdh1* subcluster via *Klf5*. *Tead4* and *Esrrb* act cooperatively with other transcription factors (e.g., *EOMES, Sox2, Tfap2C*) to established trophoblast lineage and maintain self‐renewal of trophoblast stem cells (TSCs). *Gcm1* is expressed in chorionic TSCs, and its expression is downregulated with TSC differentiation, and confined to the sites where branching of the chorionic epithelium initiates, and *Gcm1* is required for syncytiotrophoblast differentiation. Expression of selected genes involved in cell differentiation, EMT/adhesion, and migration was verified by qPCR (Figure 5D) and by immunostaining of Ki67, ZO‐1, and Vam antibody (Fugure5 E‐N). Increased expression of genes and protein of multipotency and cell‐cell adhesion (*Klf5, Cdh1, Esrrb, Tead4, Gcm1*, and ZO‐1), decreased expression genes and proteins of pro‐differentiation, EMT and morphogenesis (e.g., *TGFβ, BMP4, Wnt11, Snai2, Twist1/2, Tbx4*, Ki67, and Vcam) in *Isl1* mutant placenta suggested impaired trophoblast epithelial differentiation and villous morphogenesis.

We examined chorionic TSC proliferation and epithelial cell‐cell adhesion by immunostaining with antibodies to Ki67 and ZO‐1. In control placenta, chorionic epithelial cell proliferation was downregulated in the central areas of the placenta, especially at the sites of chorioallantoic attachment (Figure 5E). However, in *Isl1* mutants, proliferation of chorionic epithelial and allantoic mesenchymal cells was significantly increased (Figure 5F, F’). Similarly, ZO‐1 expression in control chorionic epithelium appeared to be discontinued and downregulated at the sites of chorioallantoic attachment, consistent with remodeling of the epithelial adherens junctions and initiation of chorionic villous morphogenesis (Figure 5G, G’). However, in *Isl1* mutants, ZO‐1 expression remained to be continuous across the entire epithelial layer, suggesting epithelial cell‐cell adhesions remained intact (Figure 5H, H’), which is consistent with our RNA‐seq result showing increased mRNA expression of cell junctional genes (e.g., *Cdh1, Afdn*) (Figure 5D). Since Isl1 is not expressed in chorionic trophoblasts, these results suggested that signals from *Isl1* mutant allantois failed to suppress trophoblast cell proliferation and disassemble cell adhesion complex, and initiate villous formation. Vcam1 is expressed in the distal allantois and is essential for chorioallantoic fusion. Ablation of Vcam1 causes chorioallantoic fusion defect and superficial vascular penetration into the chorion.^[^ ^23^, ^24^ ^]^ Similarly, we found that Vcam1 was markedly reduced in *Isl1* mutant allantois that may account for the observed fusion defects in *Isl1* mutants (Figure 5J, J’). Statical analysis showed that comparing to that of controls, changes of Ki67, ZO‐1, and Vcam in the mutants were significantly different (Figure 5K–M).

A substantial number of DEGs in *Isl1* mutants have been shown to play a key role in extraembryonic development, and alterations in these genes have been associated with abnormal extraembryonic tissue morphogenesis (Table S3, Supporting Information). We imported up and down‐regulated DEGs involved in extraembryonic tissue morphogenesis into String database to identify the interactions between networks of the up and downregulated DEGs (Figure 5A–C). The most outstanding interfaces between up and downregulated DEG network is the connection between the core network of transcriptional and signaling pathways (Figure S4, Supporting Information) (such as downregulated: *Bmp4, Fgf8, Tgfβ1, Pdgfrβ, Eng, Ntp1*, and *Lmo2*) and the sub‐networks of the epithelial cell development (red) (upregulated: *Cdh1, Itgav, Afdn*) and chorionic trophoblast development (upregulated: *Tead4, Gcm1*, and *Esrrb*). These analyses suggested that, during chorioallantoic fusion and labyrinth formation, combinatory actions of the major signaling pathways, genetic and epigenetic factors regulate the epithelial and endothelial gene programs and promote trophoblast differentiation, villous morphogenesis, and placenta blood vessel formation.

### 2.6 Isl1 Directly Regulates Genes for Chorioallantois Development According to CUT&Tag Results

**Figure 6:**
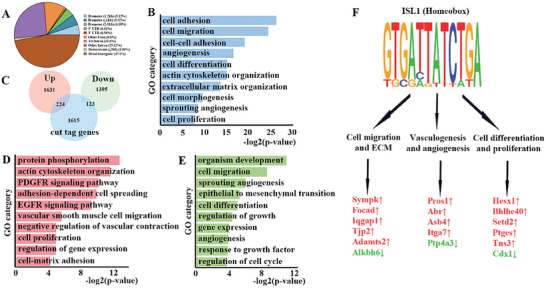
ISL1 directly regulates genes for placental development. A) Genome‐wide distribution of ISL1 cut tag peaks mapped relative to their nearest TSS (transcription start site). Annotation indicates the positions of peaks are in TTS (transcription termination site, defined as from −100 bp to +1Kb), exon, 5′UTR, 3′UTR, intronic or intergenic. B) GO analysis of genes associated with ISL1 cut tag peaks (top 10 categories are shown). C) Intersection of ISL1 cut tag and ISL1 mutant RNA‐seq datasets at E8.75 (differentially expressed genes) revealed 347 direct downstream targets of ISL1 (224 upregulated and 123 downregulated) in placenta. D,E) Enriched GO terms of direct targets of ISL1 upregulated D) and downregulated E) in placenta (top 10 categories are shown). F) Top motifs enriched in ISL1‐bound regions and promoter genes binding with ISL1 in RNA‐seq at E8.75.

To explore direct targets of Isl1 that could account for its role in chorioallantoic, we performed CUT&Tag analysis on wild type chorioallantoic cells using Isl1 antibody. Our analyses uncovered 5509 significant binding peaks for Isl1, mainly located at intergenic or intronic regions (**Figure** **6**A; Table S4, Supporting Information). Functional annotation according to the nearest transcription start site (TSS) includes those related to cell biological behavior and vessel development, consistent with the role of Isl1 in chorioallantois morphogenesis (Figure 6B). Intersection of CUT&Tag peak genes and E8.75 RNA‐seq datasets for *Isl1* mutant and control cells uncovered 347 direct targets of Isl1 downregulated (123) or upregulated (224) in *Isl1* mutant chorioallantoic (Figure 6C). GO analysis showed that the upregulated targets of *Isl1* were highly enriched in categories, including phosphorylation, cytoskeleton organization, PDGF signaling, EGF signaling, vascular smooth muscle development, and cell proliferation (Figure 6D). Categories of downregulated genes included development, cell migration, angiogenesis, EMT, cell differentiation, and cell cycle (Figure 6E). Intersection of CUT&Tag promoter genes and RNA‐seq datasets for Isl1 mutant and control cells uncovered 17 direct targets of *Isl1* in the *Isl1* mutant chorioallantoic. GO analysis showed that those genes mainly regulate cell migration, proliferation, differentiation, vasculogenesis, and angiogenesis (Figure 6F). Among those genes, *Sympk* and *Pros1* are involved in the regulation of signaling pathways associated with abortion.^[^ ^44^ ^]^

## 3 Discussion

### 3.1 Isl1 Identifies a Population of Multipotent Ex‐Mes Progenitors that Differentiate into Vascular and Mesenchymal Lineages of the Umbilicus and Fetoplacenta

The allantois is derived from the extraembryonic mesoderm; however, a detailed lineage map of the allantois's cell types and fate, and their contributions to the umbilicus and fetoplacental remain unclear. We have found that Isl1 is expressed in a major subset of Ex‐Mes and allantoic progenitors. Isl1 expression is downregulated when these progenitor cells migrate into the allantois and differentiate, suggesting that, similar to that in cardiac mesoderm,^[^ ^27^ ^]^ Is1 is a progenitor marker of the Ex‐Mes and allantois (Figure 3). Isl1 lineage tracing and coimmuostaining with the antibody marking distinct cell types of the allantois and fetoplacenta have revealed that all endothelial cells of the umbilical and labyrinth vasculatures are derived from Isl1 lineage (Tom^+^) (Figure 3). Isl1 progenitors contribute to nearly all vascular smooth muscle cells and perivascular mesenchymal cells of the umbilical cord. A number of T‐box transcription factors are expressed in the Ex‐Mes and allantois and play important roles in allantois and placenta development.^[^ ^45^, ^46^, ^47^ ^]^ Expression of Brachyury (*T*) in the Ex‐Mes and allantois ACD appears to be similar to, but earlier than Isl1, although a detailed analysis of the T lineage during Ex‐Mes and placenta morphogenesis is not reported. Tbx4 is expressed in progenitors of the Ex‐Mes and allantois, as well as differentiated allantois's cells. In contrast, the endothelial cells of the umbilicus and parts of the placenta are not derived from Tbx4 lineage,^[^ ^46^ ^]^ suggesting that Tbx4 may mark a subset of mesenchymal lineages of the allantois, whereas Isl1 lineage identify the multipotent vascular progenitors of the allantois. We have identified two distinct Isl1 progenitor populations in the allantois, namely CD34^+^ endothelial and Pdgfrβ^+^ mesenchymal progenitors. It is possible that the scattered Isl1 negative allantois's cells (about 30%, Tom‐) are of Tbx4 lineage origin. Conversely, a subset of Tbx4 lineage negative allantois's cells is noticed, which might be of Isl1 lineage origin,^[^ ^46^ ^]^ suggesting that Isl1 and Tbx4 lineages are partially complemental. Lineage derivations of fetoplacenta mesenchymal cells remains unclear, might in part are of cytotrophoblast or Ex‐Mes origins.^[^ ^48^ ^]^ We had found that there is no contribution of Isl1 lineage to the labyrinth mesenchyme, though the majority of mesenchymal cells of the umbilical cord are derived from Isl1 lineage. Tbx4 lineage strongly labels the labyrinth, but only a subset of these Tbx4 lineage labeled cells are endothelial cells, suggesting that the rest of Tbx4 lineage cells might be the mesenchymal cells.

In addition, previous study has shown that Isl1 is expressed in the amnion, and signals modulated by Isl1 from the amnion is required for embryonic mesoderm development.^[^ ^31^ ^]^ We have confirmed that Isl1 labels a substantial subset of mesenchymal cells in the amnion membranes closely associated with umbilicus (a amnion region) that has been reported to be enriched in mesenchymal stem cells.^[^ ^49^ ^]^ Therefore, a better understanding of the molecular mechanisms regulating Isl1 labeled differentiation and diversification may have a significant impact on regenerative application using cells from the perinatal extraembryonic tissues, such as umbilical cords and amnion membranes.

### 3.2 Isl1 is Required for Differentiation and Diversification of the Ex‐Mes and Allantois Progenitors, by Promoting Angioblast Differentiation and Vasculogenesis, but Repressing Mesenchymal Lineage Differentiation

The underlying molecular mechanism of the differentiation and diversification of the allantoic cell lineages is poorly understood. Chorioallantoic fusion is critically dependent on proper development of each of the allantois and chorion, and the interactions between these tissues and cell types. Deletion of *Isl1* results in defects in allantois growth and vasuclogenesis. About 40% of *Isl1* mutant embryos fail to undergo chorioallantoic fusion and the remaining *Isl1* mutant embryos, whose allantois fuses with the chorion, display severe allantois and umbilical defects, and impaired labyrinth branching morphogenesis. In *Isl1* mutant allantois, the number of CD34^+^ angioblasts is significantly decreased, but intriguingly, Pdgfrβ^+^ mesenchymal cells is increased. The cell proliferation in *Isl1* mutant allantois is significantly increased while cell death is not changed, suggesting a role for Isl1 in the diversification of Isl1^+^ Ex‐Mes progenitors by promoting endothelial but repressing mesenchymal lineage differentiation. Similar to this scenario, Isl1 regulates temporal differentiation and diversification of Isl1^+^ cardiac mesoderm progenitors by promoting myocardial differentiation, but repressing endothelial differentiation^[^ ^50^ ^]^(Our unpublished data). In contrast to Isl1, deletion of brachyury (*T*) result in allantoic hypoplasia due to reduced cell proliferation and increased cell death of the allantois core, suggesting a role for T in the expansion of progenitors of the allantois. Tbx4 mutants exhibit extensive cell death in the distal allantois where vasculogenesis initiates, but no change in cell proliferation. Since Tbx4 is not expressed in endothelial lineage, the effects of Tbx4 on vasculogenesis may be non‐cell autonomous.

Consistent with the phenotypes observed in *Isl1* mutants, RNA‐seq analysis on *Isl1* mutant Ex‐Mes and allantois progenitors at E8.25 show reduced expression of genes involved in stem cell multipotency and mesoderm development (e.g., *Sox2, Hesx1, Lhx2, Mesp1, Otx2, Isl1, Tfap2a, Zic2/5, Lefty2*, and *Cdx1*), but upregulated expression of genes involved in ECM organization and related signals (e.g., *Col3a1, Lamc2, Postn, Mmp9, Smoc2, Thy‐1, Emp2, Pdpn, Cldn3/5, CD9, Lgals3, Plet1*, and *Procr*), suggesting changes in progenitor cell potency and the extracellular microenvironments that could profoundly affect stem cell differentiation. Marked increases in ECM‐related genes in *Isl1* mutant allantois suggest increased differentiation and production of mesenchymal cells, the major ECM‐producing cells, in line with increased number of Pdgfrβ^+^ mesenchymal cells but decreased CD34^+^ angioblasts observed in *Isl1* mutant allantioses. Formation of the allantois and fetoplacenta vasculature is regulated by the conserved signaling pathways that control embryonic vascular development, such as *BMP, FGF, TGFβ, Notch*, and *PDGF VEGF*, and their receptors/coreceptors. Mutation of *T* and *Tbx4* causes changes in the signaling pathways involved in mesoderm development and vasculogenesis such as *FGF, BMP, Wnt*, and *Notch*. Using a candidate gene approach, a number of genes have been identified as potential downstream genes of *Tbx4*, including ECM (*Vcan, Has2*, and *Itga5*), transcription factors (*Snai1* and *Twist*), signaling molecules (*Bmp2, Bmp7, Notch2, Jag1*, and *Wnt2*).^[^ ^51^ ^]^ However, initially at E8.25, there is no change in those conserved keys signaling pathways in *Isl1* mutant allantois progenitors, and no change in the expression of *T* and *Tbx4* in *Isl1* mutants at E8.25. However, at the stage of chorioallantoic fusion at E8.75, RNA‐seq analysis of the allantois and fetoplacenata of *Isl1* mutants has revealed significant changes in distinct sets of genes and pathways, including multiple key signaling pathways, a number of epigenic regulators, and transcription factors, including *T* and *Tbx4* and its downstream genes, suggesting a spatiotemporal role of *Isl1* in distinct aspects of Ex‐Mes and placenta morphogenesis.

### 3.3 Signals Downstream of Isl1 from the Allantois Modulate the Chorionic Epithelial Differentiation, EMT and Labyrinth Formation

Role of allantois in the induction of chorion morphogenesis and the underlying molecular mechanisms remain not fully understood. Formation of placental labyrinth depends on timely ordered events of TSC morphogenesis, including paused proliferation and initiation of differentiation, EMT, and villous formation, which is accompanied by active angiogenesis. VCAM‐1 and its counter‐receptor, alpha‐4‐integrin are essential for of the chorioallantoic fusion and subsequent labyrinth formation.^[^ ^23^, ^24^ ^]^ In *Isl1* mutants at E8.5‐8.75 when allantois first touches to and spreads over the surface of chorion plate, endothelial layer is parallel to chorion plate and fails to penetrate the chorion epithelium that appears to remain relatively intact. The expression of Vcam1 is decreased in the distal allantois of *Isl1* mutants, similar to that in *T* and *Tbx4* mutants. Consistent with this, expression of cell‐cell adhesion molecular ZO‐1 persists in *Isl1* mutant chorionic epithelium. Further RNA‐seq analysis has revealed that a number of epithelial cell‐cell adhesion genes (*Cdh1, Afdn, Klf5*, and *Krt19*) and TSC genes (*Tead4, Gcm1, Esrrb*, and *Ly6a*) is upregulated in E8.75 *Isl1* mutant, together with decreased mesenchymal genes (*Tagln, Ahnak*, and *Pitx1*) and EMT genes (*Snai2, Twist1/2, Tgfβ1, Wnt11, Lef1*, and *Tbx4*) suggesting the allantois‐chorion contact in *Isl1* mutants fail to induce chorion trophoblast stem cells to initiate EMT to form chorionic villi and labyrinth, which may in part account for the impaired penetration of Isl1^+^ endothelial cells into chorion epithelial layer.

We have observed markable changes in the expression of a number of DNA and chromatin modification enzymes that may contribute to changes in gene expression, suggesting an essential role of Isl1 upstream of a complex epigenetic pathway in extraembryonic tissue development. However, so far biological relevance and significance of those epigenetic changes remain to be investigated in the future.

In conclusion, our studies show that Isl1 identifies a major subset of multipotent extraembryonic mesodermal and allantois progenitors that contribute to a majority of the vascular and mesenchymal cells of the placenta. Isl1 is essential for the diversification and differentiation of the Ex‐Mes and allantois progenitor and their contribution to placental morphogenesis. Signals downstream of Isl1 from the allantois mediate the interaction between the allantois and chorion that are essential for the induction of chorion epithelial differentiation and labyrinth formation. Our study suggests the potential utility of Isl1 in regenerative medicine application using the stem cells derived from the placenta, umbilical cord, and amnionic membrane.

### 3.4 Underlying Signaling and Mechanism

**Figure 7:**
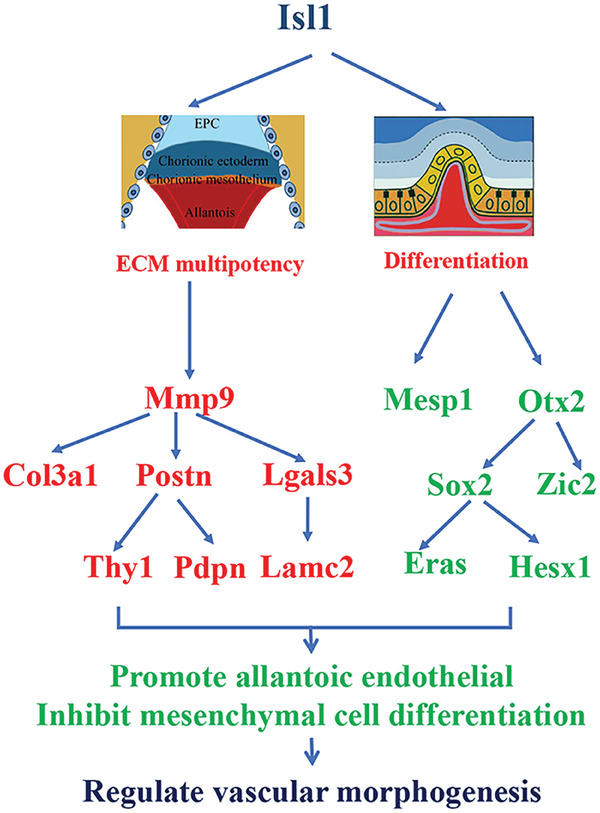
Diagram showing phenotypes observed in Isl1 mutant allantois and potential underlying mechanisms. Mmp9: Matrix metalloproteinase 9; Mesp1: Cardiovascular development mesodermal‐associated protein 1; Otx2: Orthodenticle homeobox 2; Col3a1: Collagen 3 alpha‐1; Postn: Postsynaptic protein; Lgals3: Advanced glycation end‐product receptor 3; Sox2: High mobility group box protein 2; Zic2: Zic family member 2; Thy1: Thy‐1 cell surface antigen; Pdpn: Podoplanin; Lamc2: Laminin subunit gamma‐2; Eras: ES cell expressed Ras; Hesx1: Hesx homeobox 1.

CUT&Tag and RNA seq analysis confirmed binding of *Isl1* at promoter regions of 17 genes were bound to Isl1 and their transcription was regulated by *Isl1* (Figure 6 and **Figure** **7**). Among these genes, *Sympk, Focad, Iqgap1, Tjp2, Adamts2*, and *Alkbh6* are located in focal adhesion or within fibroblast migration, involved in cellular response to growth factor stimulus, and located in several cellular components, including cortical actin cytoskeleton; focal adhesion; and lateral plasma membrane. Abnormal transcription of these genes leads to allantoic vessels (Pecam^+^cells) migrated abnormal, which further leads to abnormal vessels branch and expand (Figure 3R). *Pros1, Abr, Asb4, Itga7*, and *Ptp4a3* participate in regulation of blood vessel remodeling, vascular permeability, and vasculogenesis, including mediate cellular interactions with the extracellular matrix and other cells. Abnormal transcription of these genes causes central umbilical blood vessel. Diagram showed phenotypes observed in Isl1 mutant allantois and potential underlying mechanisms, such as, Mmp9: Matrix metalloproteinase 9; Mesp1: Cardiovascular development mesodermal‐associated protein 1; Otx2: Orthodenticle homeobox 2; Col3a1: Collagen 3 alpha‐1; Postn: Postsynaptic protein; Lgals3: Advanced glycation end‐product receptor 3; Sox2: High mobility group box protein 2; Zic2: Zic family member 2; Thy1: Thy‐1 cell surface antigen; Pdpn: Podoplanin; Lamc2: Laminin subunit gamma‐2; Eras: ES cell expressed Ras; Hesx1: Hesx homeobox 1.

## 4 Experimental Section

### Animal Models

To generate *Isl1* knock‐out mice (*Isl1* Cre^+/−^; *Isl1* ^f/+^) with Black Swiss background, *Isl1* Cre^+/−^ females and *Isl1* ^f/+^ males were closed with Rosa‐LacZ^[^ ^52^ ^]^ or Rosa‐tdTomato background^[^ ^53^ ^]^ in order to trace the lineage. *Isl1* nuclear lacZ knockin (Isl1‐LacZ), *Isl1*‐Cre knockin mouse line, and *Isl1* floxed mice line were generated as described previously.^[^ ^32^ ^]^ Mice were housed in the Laboratory Animal Facility at Tongji University. All animal experiments were performed according to the guidelines for the Care and Use of Laboratory Animals (Ministry of Health, China, 1998), and monitored by the Institutional Animal Care and Use Committee of Tongji University School of Medicine, the number of the ethical approval for animal experiments is SYXK 2020‐0002.

### Histological Analysis

The day of a vaginal plug after mating first appeared was considered as day 0.5 of gestation. Pregnant females were euthanized at different stages of gestation, embryos and placenta were dissected and fixed with 4% paraformaldehyde (PFA). Embryos (E8.25, E8.75) were stained with X‐gal solution consisting of 5 mm K_4_Fe (CN)_6_, 5 mm K_3_Fe (CN)_6_, 2 mm MgCl_2_, 0.01% NP‐40, 0.1% deoxycholate, and 0.1% X‐gal in PBS, for further analysis, embryos were paraffin‐embedded and sectioned as described previously.^[^ ^32^ ^]^ Paraffin‐embed sections were cut and stained with hematoxylin and eosin.

X‐gal staining, immunohistochemistry, cell proliferation, and apoptosis assays: Embryos were harvested and fixed for 0.5–2 h in 4% PFA. Samples were stained overnight at 37 °C in X‐gal substrate solution. For high‐resolution analysis of beta‐galactosidase activity, embryos were paraffin‐embedded and sectioned as described previously.^[^ ^32^ ^]^

For immunostaining, samples were fixed for 3 h overnight in 4% PFA, and 10 µm cryosections were cut and incubated with primary antibodies overnight at 4 °C. The following primary antibodies were used: Pecam (1:100; Vector laboratories, VP‐C344), α‐SM‐actin (1:400; Abcam, ab5694), Isl1 (1:500; DSHB, 39.4D5), CD34(1:400; Abcam, ab81289), Pdgfrβ (1:200; Abcam, ab32570), Ki67 (1:200; Abcam, ab15580), ZO‐1 (1:200; Invitrogen, 402 200), Vcam1 (1:400; Abcam, ab134047). Laminin (1:400; Abcam, ab11575), Fibronectin (1:400; Abcam, ab2413). After washing with 0.25% TritonX‐100 in PBS, sections were incubated with secondary antibodies for 2 h, and analyzed under the fluorescence microscopy (Leica).

To detect proliferation, the cryosections were incubated with an antibody to phosphor‐histone H3 (Ser10) (1:100; Upstate06‐570) and Ki67 (1:200; Abcam, ab15580). To observe cell death, the antibody to cleaved caspase 3 (Asp175) (1:400; Cell Signal, 9661s) was applied. To count positive cells, all sections of individual allantois or every third placental samples was stained, and positive cells were counted. The total number of the positive cells (Ph3^+^ and Ki67^+^ or Casp‐3^+^) and Tom^+^ cells (allantois) or DAPI nucleus (placenta) within the allantois and placenta were counted and expressed as percentage of total positive cells versus Tom^+^ or DAPI cells. Three samples per genotype were analyzed.

### RNA‐Seq and qPCR

RNA‐seq analyses with three biological repeats were performed as described^[^ ^30^ ^]^ using the allantoises of *Isl1* mutants (*Isl1* ^Cre/f^; Rosa‐Tomato) and control littermates (*Isl1* ^Cre/+^; Rosa‐Tomato) at E8.0‐8.25, and the allantoises and fetoplacentas at E8.75 before and when the chorion‐allantois fusion takes place respectively. Samples were dissected under fluorescence microscope. The RNA‐seq datasets were available from the Gene Expressing Omnibus database (http://www.ncbi.nlm.nih.gov/geo/, NCBI GEO submission number: GSE247184) under the accession number. Briefly, total RNA (100 ng) was incubated with Oligo(dT) magnetic beads to isolated mRNA. RNA‐seq libraries were prepared with the SMARTer cDNA library construction kit (Clontech) and sequenced using Illumina HiSeqTM 2000 (BGI). The differentially expressed genes were identified by FPKM ≥ 1 in either one of the two conditions, | the change of FPKM | ≥1.3–1.5 fold and *p* < 0.05. GO enrichment analysis was performed by DAVID (v6.7) gene annotation tool, and the protein‐protein interaction (PPI) network and functional enrichment analysis were performed with STRING analysis tool.

For qPCR, total RNAs were isolated with the RNeasy Micro kit (Qiagen) following the manufacturer's instructions. cDNA synthesis was performed using SuperScript II Reverse Transcriptase Kit (Invitrogen, Cat #18 064 014). qPCR was performed using Power SYBR Green PCR Master Mix (Life Technologies, Cat # 4 367 659), and the primers were used as listed (**Table** **2**).

**Table 2:** q‐PCR PRIMERS.

Gene	Forward primer	Reverse primer
Emp2	TGGTGATTCTTGCCTTCATCATT	TGATCTCGGTACAGTTTGTGCT
Adm	CACCCTGATGTTATTGGGTTCA	TTAGCGCCCACTTATTCCACT
Prl2a1	CTCCTGGTGTCTAACCTGCTT	GCAGTGAAGAGTTCAAAGGCT
Prl2c2	ATCAGGAGCCATGATTTTGGATG	CGGACTGCGTTGATCTTTTTCTT
Prl2c3	GAATGTTGCCTCATTTCC	CTGGCTTGTTCCTTGTTT
Prl3d1	GGTGTCAAGCCTACTCCTTTG	GTATTATGGAGCAGTTCAGCCAA
Prl7a1	GTCTTTCACTCAACCATGCTCC	CAGTCCCTTGATGGATAGTGGA
Mmp9	CTGGACAGCCAGACACTAAAG	CTCGCGGCAAGTCTTCAGAG
Col3a1	CTGTAACATGGAAACTGGGGAAA	CCATAGCTGAACTGAAAACCACC
Adamts2	ACGCCTTTTCTACAACCTCAC	GCCAGCCCATCACAGTTACT
Postn	CACGGCATGGTTATTCCTTCA	TCAGGACACGGTCAATGACAT
Smoc2	CCCAAGCTCCCCTCAGAAG	GCCACACACCTGGACACAT
Lgals3	AGACAGCTTTTCGCTTAACGA	GGGTAGGCACTAGGAGGAGC
Plet1	AACGATTCAGTCAGTGCCGT	TGACTTTGAGGCTGTGCGAT
Vsir	GGAACCCTGCTCCTTGCTAT	GCTGCAATGTGAAGTTGCGT
Lamc2	CAGACACGGGAGATTGCTACT	CCACGTTCCCCAAAGGGAT
Pdpn	ACCGTGCCAGTGTTGTTCTG	AGCACCTGTGGTTGTTATTTTGT
Cldn3	ACCAACTGCGTACAAGACGAG	CGGGCACCAACGGGTTATAG
Sox2	GCGGAGTGGAAACTTTTGTCC	CGGGAAGCGTGTACTTATCCTT
Mesp1	GTCACTCGGTCCTGGTTTAAG	ACGATGGGTCCCACGATTCT
Fzd5	CCAAACCTACGCTCCCAGG	CGCACCTTGTTGTAGAGTGG
Hesx1	AACGTCAGTAAGACCCCACAG	GTTCTGGGTGAAAGCGGTTC
ISL1	ATGATGGTGGTTTACAGGCTAAC	TCGATGCTACTTCACTGCCAG
Tie1	TTTTCTTGGCCTCTCATGTTGG	CGCACGATGCGATCATCCTT
Tek	CTGGAGGTTACTCAAGATGTGAC	TCCGTATCCTTATAGCCTGTCC
Foxf1	CGCTCAACGAGTGCTTCATC	ATTCATCATGCCCAAGCCGC
Hhex	CGGACGGTGAACGACTACAC	CGTTGGAGAACCTCACTTGAC
Hey1	GCCCTGGCTATGGACTATCG	CGCTGGGATGCGTAGTTGT
Sox18	CCTGTCACCAACGTCTCGC	GCAACTCGTCGGCAGTTTG
Aplnr	CAGACGCCTCGGAAAATGG	CAGCGATGGTTTGGGCAATG
Has2	TGTGAGAGGTTTCTATGTGTCCT	ACCGTACAGTCCAAATGAGAAGT
Gcm1	AGAGATACTGAGCTGGGACATT	CTGTCGTCCGAGCTGTAGATG
Ly6a	AGGAGGCAGCAGTTATTGTGG	CGTTGACCTTAGTACCCAGGA
Esrrb	AACCGAATGTCGTCCGAAGAC	GTGGCTGAGGGCATCAATG
Tpbpa	TTCCTAGTCATCCTATGCCTGG	GGTCATTTTCGCTACTGTGAAGT
Prl3b1	CACCAGACAACATCGGAAGAC	TGACAGCAGAGTATCAGGTACA
Pou5f1	CACCATCTGTCGCTTCGAGG	AGGGTCTCCGATTTGCATATCT
Cdx1	GGACGCCCTACGAATGGATG	GTACCGGCTGTAGTGAAACTC
Gata6	TTGCTCCGGTAACAGCAGTG	GTGGTCGCTTGTGTAGAAGGA
Tgfb1	CTCCCGTGGCTTCTAGTGC	GCCTTAGTTTGGACAGGATCTG
Mdk	TGGAGCCGACTGCAAATACAA	GGCTTAGTCACGCGGATGG
Bambi	GATCGCCACTCCAGCTACTTC	GCAGGCACTAAGCTCAGACTT
Eng	CCCTCTGCCCATTACCCTG	GTAAACGTCACCTCACCCCTT
Snai2	TGGTCAAGAAACATTTCAACGCC	GGTGAGGATCTCTGGTTTTGGTA
Bmp4	TTCCTGGTAACCGAATGCTGA	CCTGAATCTCGGCGACTTTTT
Wnt11	GCTGGCACTGTCCAAGACTC	CTCCCGTGTACCTCTCTCCA
Wnt5b	TCCTGGTGGTCACTAGCTCTG	TGCTCCTGATACAACTGACACA
Lef1	AACGAGTCCGAAATCATCCCA	GCCAGAGTAACTGGAGTAGGA
Ramp2	GGAGTCCCTGAACCAATCTCT	GCAACTCTTGTACTCATACCAGC
Vegfb	GCCAGACAGGGTTGCCATAC	GGAGTGGGATGGATGATGTCAG
Vegfc	GAGGTCAAGGCTTTTGAAGGC	CTGTCCTGGTATTGAGGGTGG
Rspo3	ATGCACTTGCGACTGATTTCT	GCAGCCTTGACTGACATTAGGAT
Ecscr	ATGCTTCGAGACATTTCTCTGG	TGTCGTAGGTTGAGAGCTGTAG
Emcn	CCTTTTGTCCAACAGTCTCTGC	GACACGATGCCTGGTATTGTG
Rhoj	CGGCTGCAATGGACATGAG	GGCACGTATTCCTCTGGGAAG
Esm1	CTGGAGCGCCAAATATGCG	TGAGACTGTACGGTAGCAGGT
Cxcl12	TGCATCAGTGACGGTAAACCA	TTCTTCAGCCGTGCAACAATC
Fgf8	AGGGGAAGCTAATTGCCAAGA	CCTTGCGGGTAAAGGCCAT
Nrp1	GACAAATGTGGCGGGACCATA	TGGATTAGCCATTCACACTTCTC
Nrarp	AAGCTGTTGGTCAAGTTCGGA	CGCACACCGAGGTAGTTGG
Twist1	GGACAAGCTGAGCAAGATTCA	CGGAGAAGGCGTAGCTGAG
Twist2	CGCTACAGCAAGAAATCGAGC	GCTGAGCTTGTCAGAGGGG
Tbx2	CCGATGACTGCCGCTATAAGT	CCATCCACTGTTCCCCTGT
Tbx4	TCCCCAGCTACAAGGTAAAAGT	ACCATCCATTTGTTGTCACAGAA
Tead4	GCTCTGGATGTGTGTGGAGTTCTCG	TTGGGCTTGACTGGCTGATGTG
Cdh1	GGTCATCAGTGTGCTCACCTCT	GCTGTTGTGCTCAAGCCTTCAC
Afdn	TGCCAGCCTTTCTGGATGATCC	CTGGATGGTCAAGGCAGCATTG
Otx2	TATCTAAAGCAACCGCCTTACG	GCCCTAGTAAATGTCGTCCTCTC
Zic2	GGGGCACACGAACTGTCTC	CCGGGTGGAATTAAAGGGAGG
Eras	AGTCTAGCATCTTGGACCTGAG	TGAGAGCACTTTTACCAACACC
Sparcl1	GGCAATCCCGACAAGTACAAG	TGTAGCGTCTTCCGGTGTCA
Thy1	GCTCTCAGTCTTGCAGGTGTC	CAGGCGAAGGTTTTGGTTCA
Col26a1	AGAGCGGTGCAAAATGAAGC	CCTGTAACTCACGAGGTTGGC

### CUT&Tag

CUT&Tag analysis was performed as described^[^ ^54^ ^]^ using the allantoises at E8.75 when the chorion‐allantois fusion takes place. Briefly, samples were dissected under fluorescence microscope. DNA library was constructed using Hyperactive Universal CUT&Tag Assay Kit for Illumina Pro (TD904, Vazyme). Sample test results showed that peak size was 413 bp and qPCR results showed that DNA concentration was 17.86 nmol L^−1^. The sequencing data was evaluated for data quality using FastQC software. Then trimming was used to cut off the sequencing joints and low‐quality fragments of the sequencing data. After that, Burrows Wheeler Aligner was used to compare and analyze according to the mus musculus reference genome data. Follow‐up analysis was conducted using reads of Mapping Quality > 13 as the only reads that were compared. Repeated reads were removed and peak calling analysis was performed using MACS2 software. The results showed that narrow peak count was 50 746 and summits count was 50 878. 250 bp (total 500 bp) sequence information of upstream and downstream peaks were used, and used Homer software to identify conservative sequence features of peak enrichment locations. ChIPseeker software was used to calculate the distribution of peak in each functional area. GO‐bp enrichment analysis were performed by DAVID (v6.7) gene annotation tool.

### Statistical Analysis

All experiments were performed for at least three independent times and respective data were used for statistical analyses. Data were presented as mean  ±  SD, and a two‐tailed *t*‐test was used for two‐group comparisons. Differences were considered statistically significant at a value of *p* < 0.05.

### Underlying Mechanism

notably, CUT&Tag and RNA seq analysis confirmed binding of *Isl1* at promoter regions of 17 genes were bound to Isl1, and their transcription was regulated by *Isl1* (Figure 6 and Figure 7). Among these genes, *Sympk, Focad, Iqgap1, Tjp2, Adamts2*, and *Alkbh6* were located in focal adhesion or within fibroblast migration, involved in cellular response to growth factor stimulus, and located in several cellular components, including cortical actin cytoskeleton; focal adhesion; and lateral plasma membrane. Abnormal transcription of these genes leads to allantoic vessels (Pecam^+^ cells) migrated abnormal, which further leads to abnormal vessels branch and expand (Figure 3R). *Pros1, Abr, Asb4, Itga7*, and *Ptp4a3* participate in regulation of blood vessel remodeling, vascular permeability, and vasculogenesis, including mediate cellular interactions with the extracellular matrix and other cells. Abnormal transcription of these genes causes central umbilical blood vessel remodeling impaired and allantoic vessels failed to penetrate the chorion plate according to immunostaining of Pecam, αSMA, and Ex‐Mes including Laminin and Fibronectin (Figure 3I–Q). *Hesx1, Bhlhe40, Setd2, Ptges, Tns3*, and *Cdx1* were involved in ERK1/2 cascade, Wnt signaling pathway, cell differentiation, and proliferation. Abnormal transcription levels of these genes lead to defects in allantois growth according to immunofluorescence staining of Ph3 and Ki67, and statistical analysis showed the changes of proliferation in the mutants were Significant (Figure 3S–U and Figure 5E–K).

### Statistical Analysis

All experiments were performed for four independent times and respective data were used for statistical analyses by the GraphPad Prism software. Data were presented as mean  ±  SD, and a two‐tailed *t*‐test was used for two‐group comparisons. Differences were considered statistically significant at a value of *p* < 0.05 marked by ^*^ and *p* < 0.01 marked by ^**^.

## Conflict of Interest

The authors declare no conflict of interest.

## Supporting information

Supporting Information

Supplemental Table 1

Supplemental Table 2

Supplemental Table 3

Supplemental Table 4

## Data Availability

(http://www.ncbi.nlm.nih.gov/geo/, NCBI GEO submission number: GSE247184.
