# Supplementary figures and images for: Isl Identifies the Extraembryonic Mesodermal/Allantois Progenitors and is Required for Placenta Morphogenesis and Vasculature Formation
Source: Adv Sci (Weinh). 2024 Jun 25;11(32):2400238. doi: 10.1002/advs.202400238 (PMC11348239; doi:10.1002/advs.202400238)

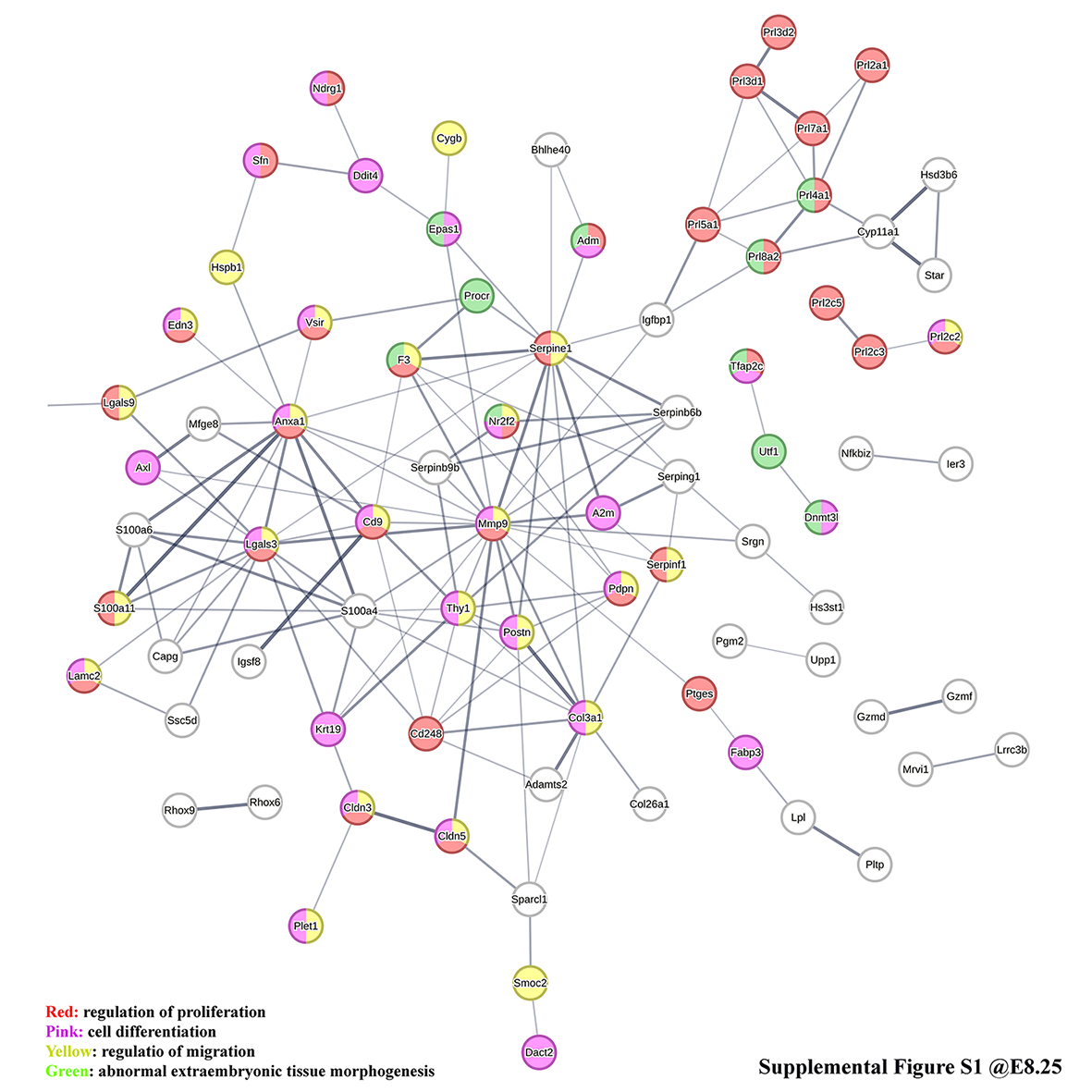


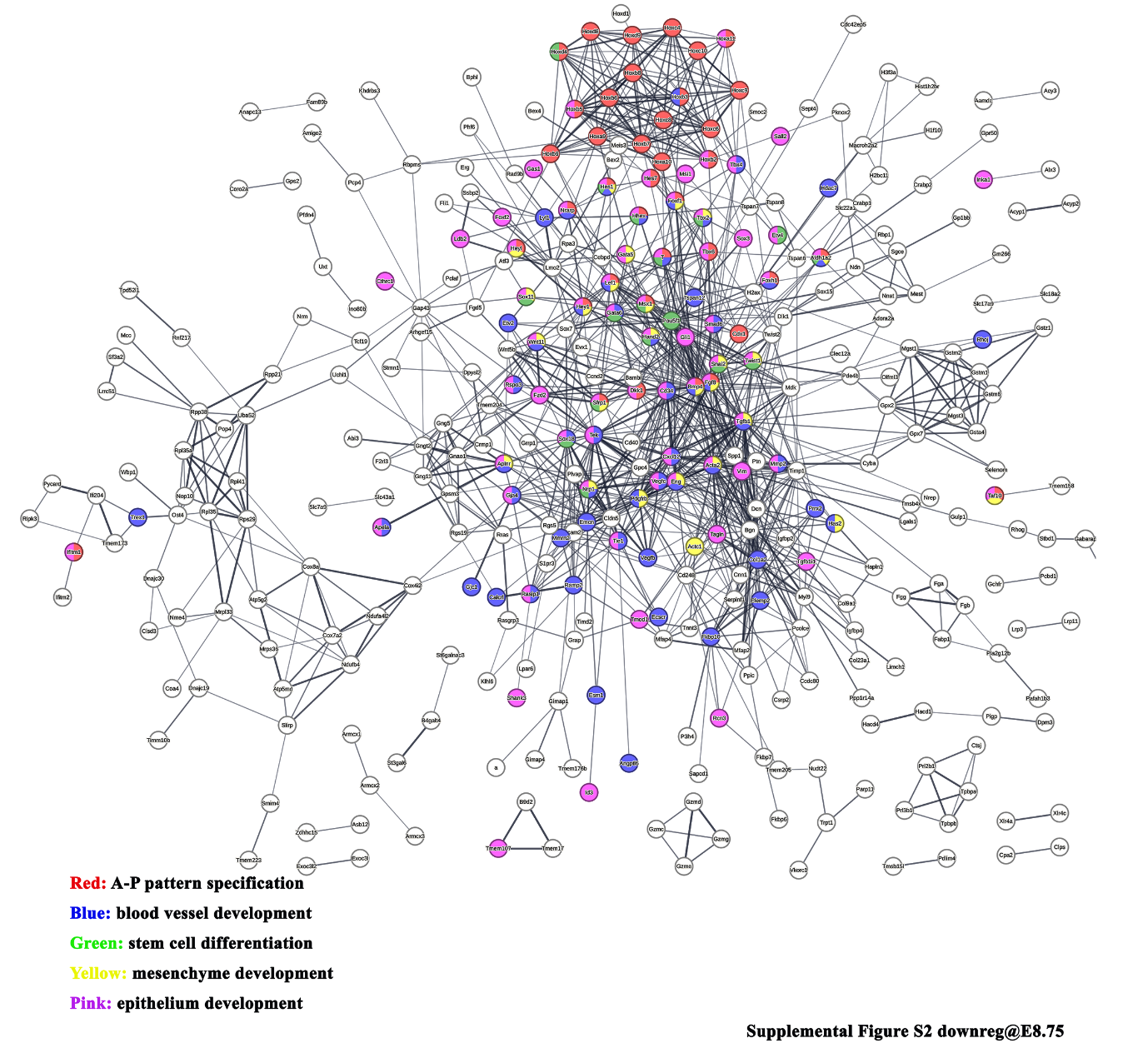


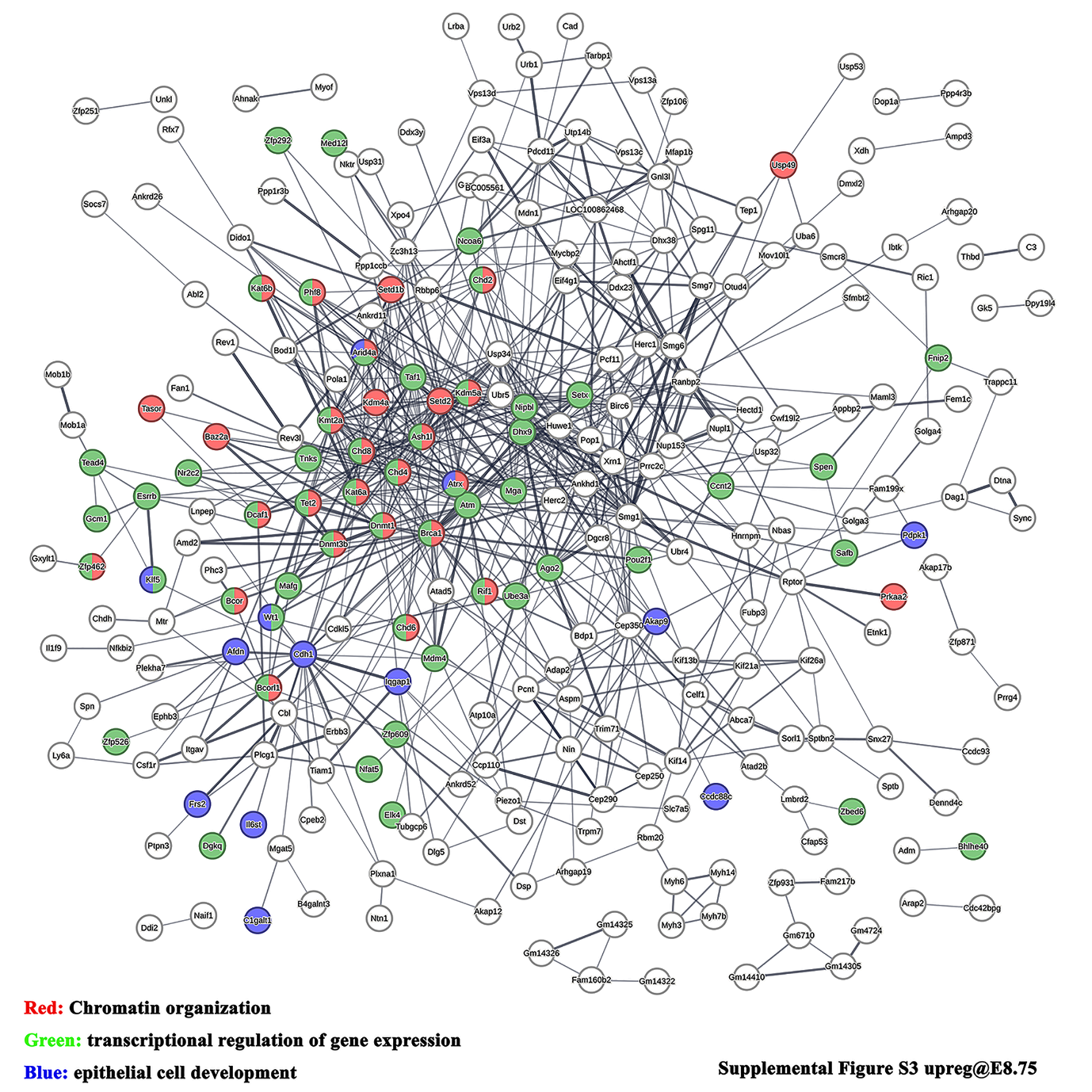


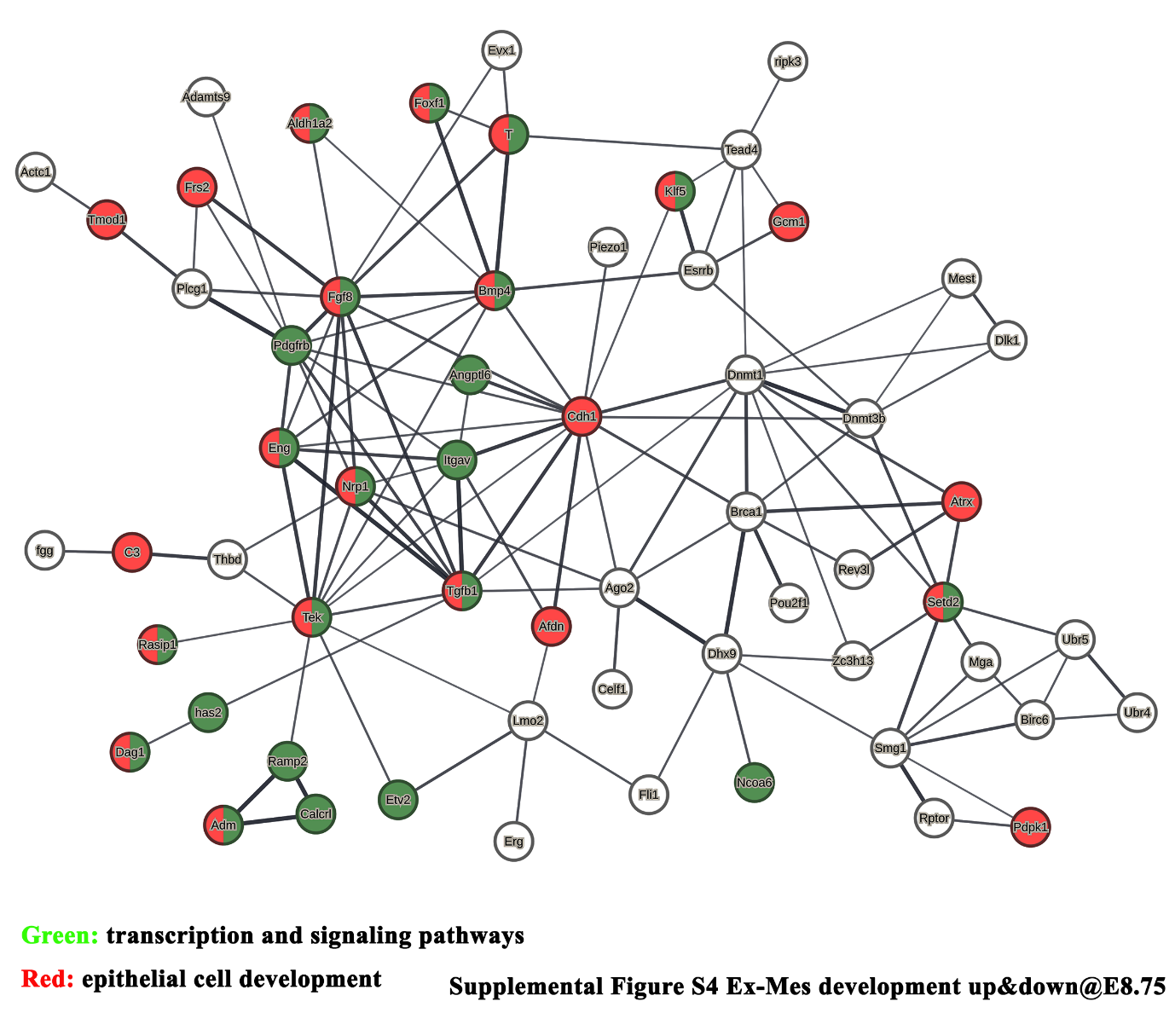

Supplement: Supplementary file 1 — Supporting Information [file ADVS-11-2400238-s004.docx]
